# Prevalence and Factors Associated with Psychological Distress among Older Adults Admitted to Hospitals After Fall Injuries in Vietnam

**DOI:** 10.3390/ijerph16224518

**Published:** 2019-11-15

**Authors:** Long Hoang Nguyen, Hai Minh Vu, Giang Thu Vu, Tung Hoang Tran, Kiet Tuan Huy Pham, Binh Thanh Nguyen, Hai Thanh Phan, Hieu Ngoc Nguyen, Bach Xuan Tran, Carl A. Latkin, Cyrus S. H. Ho, Roger C. M. Ho

**Affiliations:** 1 Center of Excellence in Behavioral Medicine, Nguyen Tat Thanh University, Ho Chi Minh City 700000, Vietnam; longnh.ph@gmail.com (L.H.N.); pcmrhcm@nus.edu.sg (R.C.M.H.); 2 Department of Trauma and Orthopaedic, Thai Binh Medical University Hospital, Thai Binh 410000, Vietnam; vuminhhai.ythaibinh@gmail.com; 3 Center of Excellence in Evidence-Based Medicine, Nguyen Tat Thanh University, Ho Chi Minh City 700000, Vietnam; giang.coentt@gmail.com; 4 Institute of Orthopaedic and Trauma Surgery, Vietnam–Germany Hospital, Hanoi 100000, Vietnam; tranhoangtung.vd@gmail.com; 5 Institute for Preventive Medicine and Public Health, Hanoi Medical University, Hanoi 100000, Vietnam; phamhuytuankiet_vkt@fpt.vn (K.T.H.P.); bach.ipmph@gmail.com (B.X.T.); 6 Department of Psychiatry, Thai Binh University of Medicine and Pharmacy, Thai Binh 410000, Vietnam; nguyenthanhbinhdhytb@yahoo.com; 7 Institute for Global Health Innovations, Duy Tan University, Da Nang 550000, Vietnam; 8 Centre of Excellence in Artificial Intelligence in Medicine, Nguyen Tat Thanh University, Ho Chi Minh City 700000, Vietnam; ngochieu.coentt@gmail.com; 9 Bloomberg School of Public Health, Johns Hopkins University, Baltimore, MD 21205, USA; carl.latkin@jhu.edu; 10 Department of Psychological Medicine, National University Hospital, Singapore 119074, Singapore; cyrushosh@gmail.com; 11 Department of Psychological Medicine, Yong Loo Lin School of Medicine, National University of Singapore, Singapore 119228, Singapore; 12 Institute for Health Innovation and Technology (iHealthtech), National University of Singapore, Singapore 119077, Singapore

**Keywords:** fall, psychological distress, Kessler 6, older adults, Vietnam

## Abstract

Although psychological distress is one of the major health issues among aging populations, little is known about how this challenge affects older patients after falls. A cross-sectional study was conducted in Thai Binh province, Vietnam, to explore the prevalence of psychological distress and associated factors among 405 older patients after falls. The 6-item Kessler Psychological Distress Scale (K6) was used to measure psychological distress. Socio-demographic and clinical characteristics were collected using a structured questionnaire. Multivariate Tobit and Logistic regressions were used to determine factors associated with psychological distress. The prevalence of psychological distress among participants was 26.2%. Patients who were alone or older had a higher likelihood of psychological distress. Patients with a history of falls in the past 12 months were more likely to suffer from psychological distress (OR = 2.87, 95%CI = 1.74; 4.72). Having two and three comorbidities was significantly associated with greater K6 scores and a higher risk of psychological distress. This study underlined a significantly high prevalence of psychological distress among older patients after falls. Providing frequent mental health monitoring, screening, treatment, and facilitating social engagements are important implications to improve the mental health of this population.

## 1. Introduction

Psychological distress later in life is an important health issue given the rapidly aging populations in all nations. This term refers to “a wide spectrum, ranging from normal feelings of vulnerability, sadness, and fears to problems that can become disabling, such as depression, anxiety, extensive worries, negative thoughts, or social isolation” [1]. Psychological distress has been confirmed to be associated with an increased risk of disability, hospitalization, and mortality [2,3,4], and decreases daily functional conditions [5] and elevates the risk of dementia [6]. Previous literature indicated that the rate of psychological distress in older adults ranged from 10.7% to 48% [7,8], and those in institutional settings experienced this mental problem more frequently than those living in their private home [9]. Interventions to prevent and reduce the onset of psychological distress among older populations are important to enhance their mental health and life quality.

Older adults experiencing falls are particularly vulnerable to mental disorders, including psychological distress. Falling is a major health issue in older adults given its commonness, as well as severe physical and psychological consequences [10]. A global report estimates that approximately one-third of older people in the community experience at least one major fall in their lifetime [11,12]. However, evidence about psychological distress in older patients after fall injuries is limited. Previous studies in developed countries revealed that 25%–30% of older patients experienced other psychological disorders such as anxiety, depression, or posttraumatic stress [13,14,15]. Factors associated with these mental disorders varied across settings, including socio-demographic characteristics (e.g., advanced age, female, low education level, and unemployment) and clinical characteristics (history of falls, volume of comorbidity, and back/chest injuries) [13,14,15]. Understanding the psychological distress patterns and their determinants in specific settings is important to develop contextualized interventions for the improvement of older adults’ mental health.

Vietnam is undergoing a remarkable increase in the aging population, from 8.9% in 2009 to reach 30% of the general population in 2050 [16,17]. Therefore, developing strategies to improve older adults’ health and well-being is prioritized in public policy agendas. However, studies about falls in this population, as well as mental disorders of older adults hospitalized after falls, have not been fully investigated. This study thus aimed to explore the prevalence of psychological distress among hospitalized older patients due to falls and to determine associated factors.

## 2. Materials and Methods

### 2.1. Study Design and Sample

Data for this study came from a cross-sectional study from seven hospitals, comprising of Thai Binh Provincial General Hospital and six district hospitals, namely Kien Xuong, Quynh Phu, Tien Hai, Thai Thuy, Dong Hung, and Hung Ha. The sample consisted of patients who were 60 years of age or older, hospitalized due to falls (inpatient or outpatient), and did not suffer cognitive impairment, which could affect their ability to answer the questionnaire. There were 430 patients who were conveniently recruited, of which, 405 patients agreed to enroll in this study (response rate 94.2%).

### 2.2. Data Collection

Fifteen-minute face-to-face interviews were performed via a structured questionnaire by trained undergraduate medical students from the Thai Binh University of Medicine and Pharmacy. Older patients were introduced briefly to the study’s purpose, as well as their benefits and rights while participating. After that, they were asked to give their written or verbal informed consent.

#### 2.2.1. Primary Outcome

This study used the Kessler Psychological Distress Scale 6 items (K6) to evaluate older patients after falls. This instrument used an inventory of 6 items to inquire about patients’ feelings over the past 30 days, including: nervous, hopeless, restless/fidgety, depression, “everything was an effort”, and feelings of worthlessness. Each question has five categories of responses, from 0 “None”, 1 “A little”, 2 “Sometimes”, 3 “Most of the time” and 4 “Always” [18]. The possible score ranges from 0 to 24, with a higher score indicating greater psychological distress. In this study, we used a score of at least 6 points as the cut-off point to determine psychological distress. The internal consistency of this instrument was accepted with a Cronbach’s alpha of 0.78.

#### 2.2.2. Covariates

We collected data about demographic characteristics including age, gender, marital status (single/having spouse/partner), living location (urban/rural), living arrangements (spouse/alone/children/others), caregiver (spouse/children/others), and smoking status (no smoking/former, smoker/irregular, smoker/regular smoker). We also measured some clinical indicators, such as other falls in the past 12 months (except the last fall causing this hospitalization), type of patient (inpatient/outpatient), and number of comorbidities.

### 2.3. Statistical Analysis

STATA version 14.0 (Stata Corp. LP, College Station, TX, United States of America) was used for data analysis. Chi-square and Mann–Whitney tests were used to compare the difference in various characteristics between those with and without psychological distress. The factors related to the Kessler score and Psychological Distress status were determined using multivariate Tobit regression and Logistic regression models. We utilized a backward stepwise selection strategy to select the models, in which variables with *p*-values of log-likelihood ratio test <0.1 were included, and those with *p*-values >0.2 were excluded. A *p*-value of less than 0.05 indicates statistical significance.

### 2.4. Ethical Approval

The study protocol was reviewed and approved by the Institutional Review Board of Thai Binh University of Medicine and Pharmacy (Code: 7641/HDDD).

## 3. Results

Among 405 patients enrolled in the study, the prevalence of psychological distress was 26.2%. Table 1 shows that the rate of psychological distress was significantly higher in patients who were female (30.0%), single (42.0%), and lived alone (47.1%) (*p* < 0.05). Psychological distress was significantly lower in patients who had spouses as caregivers (17.9%) and were regular smokers (5.0%) (*p* < 0.05). Patients with psychological distress also had a significantly higher age (mean = 75 years old, SD = 9.0) compared to those without psychological distress (mean = 70.7 years old, SD = 8.7).

**Figure 1:**
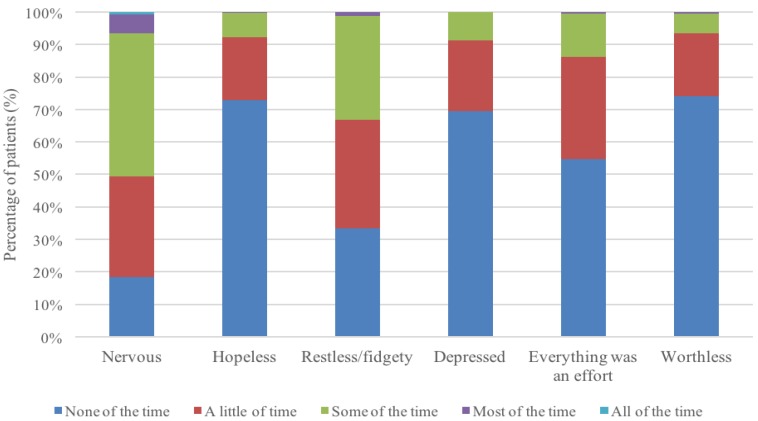
Profile of 6-item Kessler Psychological Distress Scale (K6) responses.

The mean K6-score was 4.1 (SD = 3.0). Figure 1 shows the K6 profiles of older patients hospitalized for fall injuries. Nervous, restless/fidgety, and “everything was an effort” were the three most frequently endorsed aspects of psychological distress among participants.

In Table 2, the prevalence of psychological distress among older patients with ear/hearing diseases or impairment (56.3%), lumbar spine pain/cervical spine pain (39.8%), and osteoarthritis/arthritis (32.4%) was significantly higher than those who did not have these conditions (*p* < 0.05). In addition, people suffering from falls in the last 12 months (60.4%) had higher rates of psychological distress compared to those who did not fall. The psychological distress rate increased from patients with one comorbidity (18.8%), two comorbidities (32.4%), and three or more comorbidities (50.0%). These differences were statistically significant (*p* < 0.05).

Table 3 depicts the results of multivariate regressions showing factors associated with psychological distress among older patients after falls. Only variables having a p-value of <0.2 were presented in the table. An increase in age by one year led to an increase of 3% in the risk of having psychological distress (OR = 1.03, 95%CI = 1.00; 1.06). Former smokers and regular smokers had significantly lower scores (Coef. = −1.01, 95%CI = −2.02; −0.01 and Coef. = −1.63, 95%CI = −3.14; −012) than those who never smoked. Notably, patients who were alone had a 5.47-times higher likelihood of psychological distress compared to those living with a spouse (OR = 5.47, 95%CI = 1.86; 16.10).

Patients having a history of falls in the past 12 months were 2.87-times more likely to suffer from psychological distress (OR = 2.87, 95%CI = 1.74; 4.72). Moreover, they had a higher K6 score that was 1.47 points higher compared to those who did not have a history of falls (Coef. = 1.47, 95%CI = 0.82; 2.11). Having two and three comorbidities was significantly associated with greater K6 scores, as well as higher risk of psychological distress, than those without any comorbidities.

## 4. Discussion

To the best of our knowledge, this is among one of the first studies examining the mental health of older patients hospitalized after fall injuries. Our study revealed a high prevalence of psychological distress in this population. Moreover, we found that greater age, higher number of comorbidities, having a history of falls, and living alone increased the risk of psychological distress in these patients. These findings are critical for developing further interventions to improve mental health among older adults hospitalized due to fall injuries in Vietnam.

The prevalence of psychological distress in our sample was consistent with the rate of other mental disorders (e.g., anxiety or posttraumatic disorder) in other developed countries such as the United States (27%) [13], the United Kingdom (25%) [14], and France (26%) [15]. Small variances might be due to the difference in setting, the severity of injuries, and measurement instruments. However, our study echoes previous work that found this issue is high among patients after falls. Therefore, mental disorders such as psychological distress should be considered when delivering treatment and care for older patients after falls. Nervous and restless/fidgety were two major psychological symptoms attributable to the psychological distress among our patients. We observed that patients were worried about their physical conditions and often wondered whether they could recover or not. This implies that the psychological distress symptoms might be reduced if patients were informed of the treatment progress or encouraged regularly by physicians.

In this study, we found that increasing age increased the risk of psychological distress among this sample of older patients. This result might contradict some previous studies which showed that in older adults, people with a lower age were more likely to suffer from psychological distress than others [15,19,20]. We hypothesize that this relationship can be explained by the fact that in our sample, people with an older age may have had a history of falls in the past 12 months, as well as a higher number of comorbidities (data not shown). Indeed, in regression models, these two factors were found to be significantly associated with psychological distress. This finding was in line with prior studies [13,14,21,22] which indicated that a greater burden of medical conditions, as well as the fear of falling due to prior falls, were related to posttraumatic stress symptoms among hospitalized older patients after falls. Moreover, our study was in line with other previous studies that reported that an increasing number of chronic comorbidities could elevate the risk of psychological issues in other, older adult populations [23,24]. Indeed, experiencing more diseases possibly affects a patients’ recovery process and may prolong the length of hospitalization [25]—a predictor of psychological distress [26].

Findings of this study clearly revealed that loneliness was significantly associated with an increased risk of psychological distress among older people after falls compared to those living with a spouse/partner. In prior literature, the effects of living alone are controversial. A few studies showed that among older adults, loneliness could result in better psychological health [27,28], while many studies reported that it could increase vulnerability to poor mental health, as well as increase the risk of depression and anxiety [27,29,30]. This relationship depends heavily on whether individuals are well connected with their community or are socially isolated and lack social supports [20]. Meanwhile, most of the studies reach a consensus that older people living with a spouse/partner had the best mental health outcomes compared to those living with other people, especially among older people with limited functional conditions [27,28,29]. Interestingly, we found that older patients having children as caregivers had higher scores of psychological distress than those having a spouse/partner as their caregiver. This might be explained by older people with disabilities viewing themselves as burdensome to their children [30], potentially increasing the risk of mental disorders.

Findings of this study suggest several important implications. First, given the high prevalence of psychological distress among older patients hospitalized after falls, screening and monitoring of mental health conditions should be routinely performed in clinical settings. Second, mental health counseling services should be provided to these patients to improve their psychological condition. Third, family members should be offered educational sessions to enhance their understanding of psychological distress, as well as information on how to improve patients’ physical and mental health. Moreover, lonely older patients should be encouraged to get involved in social activities to increase interactions and build friendship networks.

This study contained several limitations. First, we did not measure the severity of fall injuries which might potentially influence the risk of psychological distress in older patients experiencing falls. Second, a cross-sectional design was used in this study; therefore, causal relationships between psychological distress and its associated factors could not be examined. Additional longitudinal studies should be implemented to fill this gap. Third, a convenient sampling method restricted the generalizability of our results to other hospitals in Vietnam. Finally, the K6 instrument has not been validated elsewhere to measure the psychological distress in older patients hospitalized due to fall injuries. Further validation studies in this population are warranted.

## 5. Conclusions

This study complements the current literature on the significantly high prevalence of psychological distress among older adults hospitalized due to fall injuries. Providing frequent mental health monitoring, screening, treatment, and facilitating social engagements are important implications to improve the mental health of this population.

## Figures and Tables

**Table 1:** Socio-demographic and behavior characteristics of respondents.

Characteristic	Not Having Psychological Distress		Having Psychological Distress		Total		*p*-Value
	n	%	n	%	n	%	
**Total**	299	73.8	106	26.2	405	100.0	
**Gender**							
Male	129	79.6	33	20.4	162	40.0	0.03
Female	170	70.0	73	30.0	243	60.0	
**Marital status**							
Single	76	58.0	55	42.0	131	32.3	<0.01
Having spouse/partner	223	81.4	51	18.6	274	67.7	
**Location**							
Urban	26	81.3	6	18.8	32	7.9	0.32
Rural	273	73.2	100	26.8	373	92.1	
**Living arrangements**							
Spouse	193	82.1	42	17.9	235	58.0	0.00
Alone	9	52.9	8	47.1	17	4.2	
Children	75	61.5	47	38.5	122	30.1	
Others	22	71.0	9	29.0	31	7.7	
**Caregiver**							
Spouse	174	82.1	38	17.9	212	52.4	<0.01
Children	102	64.6	56	35.4	158	39.0	
Other	23	65.7	12	34.3	35	8.6	
**Smoking**							
No smoking	230	70.8	95	29.2	325	80.3	0.03
Former smoker	39	84.8	7	15.2	46	11.4	
Irregular smoker	11	78.6	3	21.4	14	3.5	
Regular smoker	19	95.0	1	5.0	20	4.9	
	**Mean**	**SD**	**Mean**	**SD**	**Mean**	**SD**	
**Age**	70.7	8.7	75.1	9.0	71.9	9.0	<0.01

**Table 2:** Psychological distress according to comorbidity and history of falls.

Characteristic	Not Having Psychological Distress		Having Psychological Distress		Total		*p*-Value
	n	%	n	%	n	%	
**Type of patients**							
Inpatient	115	76.2	36	23.8	151	37.3	0.41
Outpatient	184	72.4	70	27.6	254	62.7	
**Hypertension**							
Yes	101	75.4	33	24.6	134	33.1	0.62
No	198	73.1	73	26.9	271	66.9	
**Heart-related diseases**							
Yes	33	64.7	18	35.3	51	12.6	0.11
No	266	75.1	88	24.9	354	87.4	
**Ear/hearing diseases**							
Yes	7	43.8	9	56.3	16	4.0	0.01
No	292	75.1	97	24.9	389	96.1	
**Lumbar spine pain/cervical spine pain**							
Yes	53	60.2	35	39.8	88	21.7	<0.01
No	246	77.6	71	22.4	317	78.3	
**Osteoarthritis/Arthritis**							
Yes	92	67.7	44	32.4	136	33.6	0.04
No	207	77.0	62	23.0	269	66.4	
**Others**							
Yes	39	66.1	20	33.9	59	14.6	0.14
No	260	75.1	86	24.9	346	85.4	
**Number of comorbidities**							
0	78	78.8	21	21.2	99	24.4	<0.01
1	130	81.3	30	18.8	160	39.5	
2	69	67.7	33	32.4	102	25.2	
3	22	50.0	22	50.0	44	10.9	
**Other falls in the past 12 months (except the last fall causing hospitalization)**							
Yes	100	33.4	64	60.4	164	40.5	<0.01
No	199	66.6	42	39.6	241	59.5	

**Table 3:** Factors associated with psychological distress among older patients after falls.

Characteristic	K6 Score		Having Psychological Distress (Yes/No)	
	Coef.	95%CI	OR	95%CI
**Age**			1.03 **	(1.00; 1.06)
**Smoking status**				
Never smoking	REF		REF	
Former smoker	−1.01 **	(−2.02; −0.01)	0.56	(0.23; 1.36)
Regular smoker	−1.63 **	(−3.14; −0.12)		
**Living arrangements**				
Spouse	REF		REF	
Alone			5.47 ***	(1.86; 16.10)
Children	1.07 ***	(0.37; 1.77)	1.75 *	(0.99; 3.07)
**Caregiver**				
Spouse	REF			
Other	1.39 **	(0.25; 2.54)		
**Fall in the past 12 months**				
No	REF		REF	
Yes	1.47 ***	(0.82; 2.11)	2.87 ***	(1.74; 4.72)
**Type of patient**				
Inpatient	REF			
Outpatient	0.54	(−0.11; 1.19)		
**Number of comorbidities**				
0	REF		REF	
1			0.90	(0.47; 1.75)
2	1.53 ***	(0.79; 2.26)	1.98 *	(1.00; 3.91)
3	1.33 **	(0.30; 2.37)	2.67 **	(1.16; 6.15)

REF: Reference; *** *p* < 0.01, ** *p* < 0.05, * *p* < 0.1.
